# Machine learning-based integration of DCE-MRI radiomics for STAT3 expression prediction and survival stratification in breast cancer

**DOI:** 10.3389/fimmu.2025.1619186

**Published:** 2025-06-25

**Authors:** Dong Pan, Cheng-Yan Zhang, Ya-Fei Wang, Shuang Liu, Xiong-Zhi Wu

**Affiliations:** ^1^ Tianjin NanKai Hospital, Tianjin Medical University, Tianjin, China; ^2^ Tianjin Key Laboratory of Acute Abdomen Disease Associated Organ Injury and ITCWM Repair, Tianjin, China; ^3^ Institute of Integrative Medicine for Acute Abdominal Diseases, Tianjin, China; ^4^ Department of Gastroenterology, Shanxi Province Cancer Hospital/Shanxi Hospital Affiliated to Cancer Hospital, Chinese Academy of Medical Sciences/Cancer Hospital Affiliated to Shanxi Medical University, Taiyuan, China; ^5^ Tianjin Medical University Cancer Institute and Hospital, National Clinical Research Center for Cancer, Tianjin, China; ^6^ Tianjin’s Clinical Research Center for Cancer, Tianjin, China

**Keywords:** breast cancer, immune microenvironment, prognosis, STAT3, machine learning

## Abstract

**Objective:**

To explore the association between signal transducer and activator of transcription 3 (STAT3) expression, tumor immune microenvironment, and overall survival (OS) in breast cancer, and to develop a non-invasive radiomics model for early risk stratification using dynamic contrast-enhanced magnetic resonance imaging (DCE-MRI).

**Methods:**

Data from 1,008 patients with breast cancer in The Cancer Genome Atlas were analyzed to evaluate the prognostic significance of STAT3 expression using Kaplan-Meier survival analysis and Cox regression models. Functional enrichment and immune cell infiltration analyses were performed to assess tumor immune microenvironment characteristics. Additionally, DCE-MRI data from 101 patients in The Cancer Imaging Archive were used to extract radiomic features from early- and delayed-phase images. A STAT3 predictive model was developed using six machine learning algorithms. Model performance was assessed using receiver operating characteristic (ROC) and related diagnostic statistical indicators.

**Results:**

Low STAT3 expression was significantly associated with poorer OS (hazard ratio [HR] = 1.927, *p* < 0.001). GSEA revealed that high STAT3 expression enhanced epithelial apoptosis and TNF-α/NFκB signaling while suppressing pro-tumorigenic pathways, which was associated with an immunosuppressive microenvironment, whereas low STAT3 correlated with T-cell exhaustion. DIA confirmed elevated STAT3 in tumor versus normal tissue (*p* < 0.05). The logistic regression-derived radiomics model for STAT3 expression prediction exhibited consistent discriminative performance, with area under curve (AUC) values of 0.861 (95% CI: 0.749 - 0.947) in the development cohort and 0.742 (95% CI: 0.588 - 0.884) in the validation cohort. High radiomics-derived scores were positively correlated with elevated STAT3 expression, longer OS (*p* = 0.034), and immune-related gene signatures indicative of a heightened immune response.

**Conclusion:**

Radiomics analysis of DCE-MRI images in this study offered a non-invasive method for predicting STAT3 expression and characterization of the tumor immune microenvironment. This approach can offer valuable insights into breast cancer prognosis and support the development of personalized therapies.

## 1 Introduction

Breast cancer remains the most prevalent malignancy and leading cause of cancer-related mortality among women worldwide (1). Although significant progress has been made in primary treatments (surgery, chemotherapy, and radiotherapy), 10%–20% of early-stage patients still experience recurrence and metastasis within five years (2). This underscores the critical need for more precise prognostic tools to enable early intervention and personalized therapy. While current prognostic markers including TNM staging, molecular subtypes, and treatment modalities (3–5) could provide valuable information, they are limited by subjectivity, poor reproducibility, and the invasive nature of tissue sampling, which may not fully represent tumor heterogeneity, highlighting the necessity for objective, non-invasive methods to enhance the accuracy of prognostic assessments (6).

Signal transducer and activator of transcription 3 (STAT3) is a key transcription factor that plays a dual role in immune regulation and tumor progression, making it a compelling target for cancer research (7, 8). Unlike immune checkpoint markers such as PD-L1, which primarily modulate T-cell activity, STAT3 drives oncogenic processes directly by promoting tumor cell survival, proliferation, and metastasis (9, 10). Its activation upregulates key mediators like cyclin D1, c-myc, and Bcl-2, enabling breast cancer progression, while also enhancing metastatic potential through matrix metalloproteinases (MMPs) (11–13). Given its central role in both immune evasion and tumor aggressiveness, STAT3 inhibition offers a broader therapeutic strategy compared to pathway-specific targets like PD-L1 (14). This study focuses on STAT3 to elucidate its tumor-intrinsic mechanisms and explore its potential as a multifaceted therapeutic target in breast cancer.

Radiomics provides a powerful framework for non-invasively linking imaging phenotypes to molecular characteristics (15). Dynamic contrast-enhanced magnetic resonance imaging (DCE-MRI), known for its superior soft tissue resolution, provides detailed and quantifiable data, making it increasingly valuable in breast cancer diagnosis and treatment. The integration of radiomics with DCE-MRI images has emerged as a transformative strategy for the non-invasive diagnosis and prognosis of breast cancer, aiding in tumor identification, characterization, staging, and treatment planning. This approach advances personalized medicine by quantifying tumor heterogeneity, offering tailored therapies, and prognostic insights However, current radiomics research primarily focuses on macroscopic tumor features, with limited investigation of underlying molecular mechanisms.

While radiogenomic correlations have been well characterized in malignancies such as lung adenocarcinoma (16), hepatocellular carcinoma (17), head and neck squamous cell carcinoma (18), glioma (19), gastrointestinal tumor (20), and pancreatic cancer (21), no study has been reported in breast cancer regarding non-invasive biomarkers for STAT3 pathway activation and its clinical implications. To address this gap, we aimed to develop a non-invasive radiomics approach using DCE-MRI to predict STAT3 expression and assess tumor immune status. Our approach integrates bioinformatics with machine learning (ML), employing six distinct classifiers to construct predictive models from early- and delayed-phase MRI radiomic features. By systematically optimizing model performance, we aim to establish a robust, imaging-based tool for prognostic stratification and personalized therapeutic decision-making in breast cancer.

## 2 Materials and methods

### 2.1 Study sample

The study incorporated multi-modal data from established public repositories. From The Cancer Genome Atlas Breast Invasive Carcinoma collection (TCGA-BRCA) (22), we obtained RNA-seq and clinical data for 1,070 breast cancer patients, applying stringent quality controls that excluded: (a) 20 samples with inadequate sequencing quality; (b) 30 patients with OS <30 days, to reduce non-cancer-related mortality bias; and (c) 12 male patients, resulting in a final cohort of 1,008 cases with complete molecular and clinical profiles. To establish a robust normal tissue reference, 80 female breast samples from GTEx (23) were acquired and integrated after batch effect correction using the “limma” package (**Additional file 1**).

**Figure 1:**
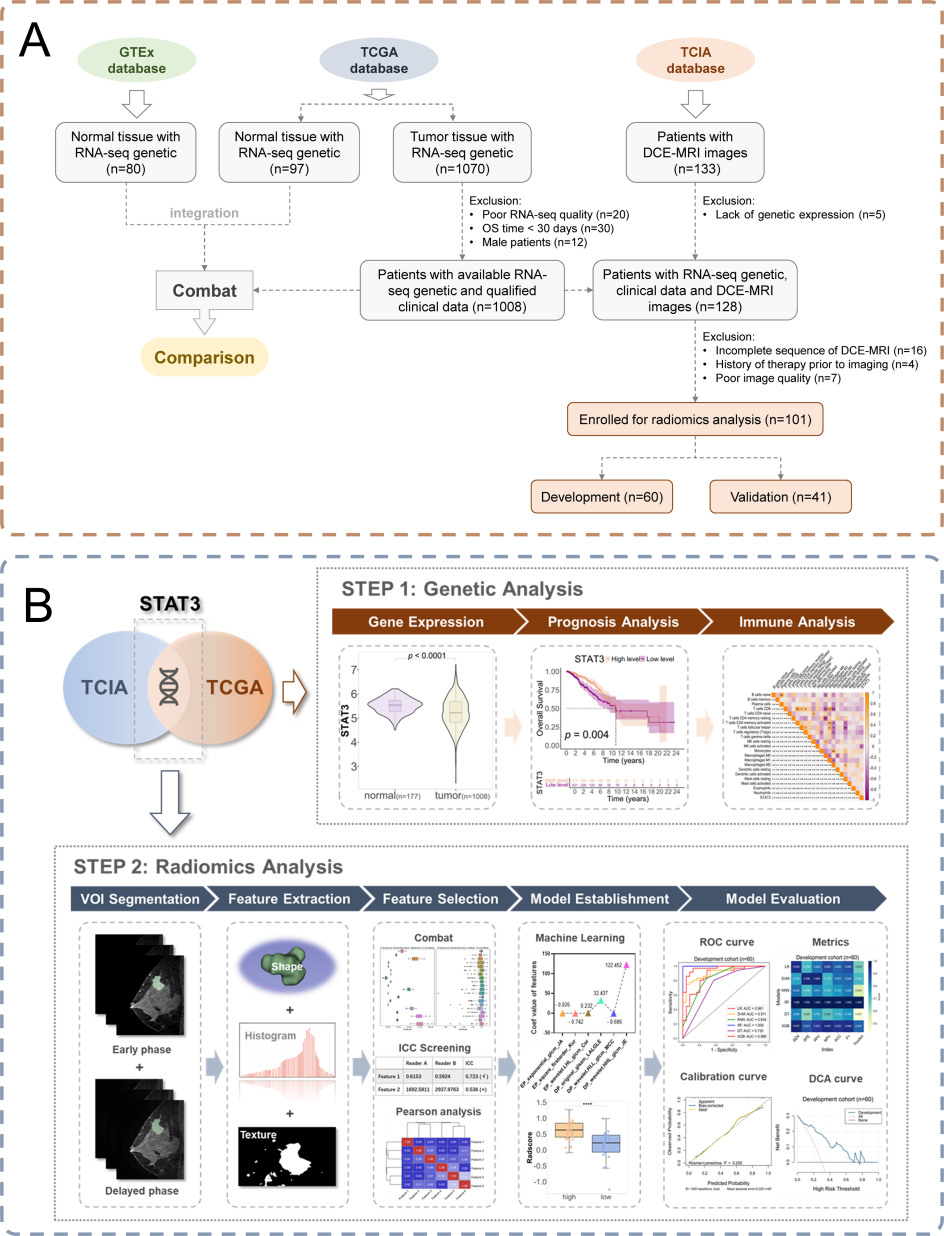
Study workflow. **(A)** Patient enrollment flow chart; **(B)** Schematic diagram of the workflow of an imaging histology study.

For radiogenomic analysis, DCE-MRI data from The Cancer Imaging Archive (TCIA) database (24) were analyzed. TCGA and TCIA breast cancer cohorts were matched using patient IDs and DICOM metadata to ensure consistent patient populations. Exclusion criteria included: (a) missing gene expression data (n = 5); (b) incomplete MRI sequences (n = 16); (c) prior treatments (n = 4); and (d) suboptimal image quality, including SNR < 20 dB, presence of motion artifact or incomplete image coverage (n = 7), yielding 101 patients with matched imaging-genomic data. All molecular profiles (including tumor/normal samples) were accessed via Xena (25), while immune-related gene sets were sourced from ImmPORT (26) (**Additional file 2**). The integrated study design is summarized in **Figure 1A**, and the follow-up research process is shown in **Figure 1B**. As this research utilized exclusively de-identified, publicly available data, institutional review board approval was waived.

### 2.2 Digital image analysis of STAT3 expression

STAT3 expression was quantified using standardized IHC with a validated anti-STAT3 antibody on FFPE tumor sections, following established protocols with appropriate controls. Digital image analysis was performed using QuPath, with tumor and stromal compartments annotated by pathologists. Expression levels were assessed through integrated and average optical density measurements. Methodological rigor was ensured through technical reproducibility testing, molecular correlation with transcriptomic data, and clinically relevant threshold determination. Complete details are provided in the Methods section.

### 2.3 Association between STAT3 expression and prognostic outcomes in patients

We employed the minimum *p*-value method to establish the optimal STAT3 expression cutoff, stratifying patients into low (n = 431) and high (n = 577) expression groups. Survival outcomes were compared using Kaplan-Meier analysis (“survminer” package) with log-rank testing (95% CIs). A 10-year landmark analysis was implemented as it represents a clinically meaningful timeframe for breast cancer outcomes while maintaining adequate statistical power, capturing both early immunoediting and late immune escape phases relevant to STAT3 biology.

### 2.4 Assessment of clinical prognostic factors through cox regression and subgroup analyses

Prognostic analyses were conducted using a two-stage approach: (1) univariate Cox regression (“survival” package) identified significant clinical risk factors, which were then (2) incorporated into multivariate models adjusting for potential confounders. Subgroup analyses evaluated STAT3-prognosis associations across key clinical strata including: age, gender, menopausal status, prior malignancy history, clinical stage at diagnosis, surgical procedures for breast carcinoma and axillary lymph nodes, tumor histological type, pathological TNM stage, and radiation therapy administration.

### 2.5 Functional analysis by Gene Set Enrichment Analysis

Gene Set Enrichment Analysis (GSEA) was performed to investigate pathway associations with STAT3 expression in breast cancer. We conducted differential pathway analysis comparing high versus low STAT3 expression groups, examining Hallmark, Kyoto Encyclopedia of Genes and Genomes (KEGG), and Gene Ontology (GO) terms. The corresponding gene sets for Hallmark, KEGG, and GO pathways were obtained from the GSEA database for this analysis. Significance of enriched pathways was determined using a nominal *p*-value < 0.05 and an FDR-adjusted *q*-value threshold of < 0.25 to account for multiple testing. Pathways meeting these criteria were considered statistically significant.

### 2.6 Immune microenvironment and treatment predictions in breast cancer

To explore the TIME, RNA-seq data from all patients were analyzed using the CIBERSORTx database (27) to evaluate immune cell infiltration. Spearman correlation analysis was used to examine the association between STAT3 expression levels and immune cell infiltration in breast cancer, providing insights into the immune landscape and its potential implications for treatment predictions.

### 2.7 Construction and evaluation of the radiomic model

An experienced radiologist performed semi-automated 3D tumor segmentation using 3D Slicer on early- and delayed-phase MRI from 101 patients, generating paired volumes of interest (VOIs) per case. Inter-reader consistency was assessed in a 30-case subset by a senior radiologist.

Image preprocessing (bias field correction, resampling) was performed using PyRadiomics (v3.01) and SimpleITK (v2.2.0). Radiomic features (first-order, morphological, texture, and high-order statistics) were extracted from segmented VOIs. To mitigate batch effects, ComBat harmonization (Batch=6) was applied using scanner model information from DICOM metadata, followed by Z-score normalization. Subsequently, patients were divided into development/validation sets (6: 4) by STAT3 expression, balanced via Chi-square/Fisher’s tests. Features were screened in the development cohort, retaining only features with ICC > 0.70. Following redundancy reduction (r > 0.90), LASSO regression with 50% discount cross-validation selected optimal features.

The predictive models were developed using six ML algorithms (LR, SVM, KNN, RF, DT, XGBoost) to generate radiomics scores via SPSS (v26). To prevent overfitting with limited samples, we optimized the scikit-learn framework and hyperparameters via grid search (detailed in **Additional file 3**). Model discrimination was assessed through receiver operating characteristic (ROC) analysis with area under curve (AUC). The Youden index-derived cutoff optimized Rad-score classification, followed by comprehensive evaluation using sensitivity, specificity, positive predictive value (PPV), negative predictive value (NPV), and F1-score. The best-performing model in the validation cohort was selected for clinical application. The optimal model identified in the validation cohort was further subjected to bootstrap resampling (n = 1,000 iterations) to evaluate its robustness. Additional validation included Hosmer-Lemeshow testing, calibration curves, and decision curve analysis (DCA) to assess clinical utility

### 2.8 Integrated analysis of radiomics, STAT3 expression, survival, and immune correlations

Our radiomics model was employed to calculate RAD-scores for patients stratified by STAT3 expression levels (high vs low) across development and validation cohorts. Using the Youden index, we established RAD-score thresholds to categorize patients into high-RAD and low-RAD groups. Survival outcomes were then analyzed through Kaplan-Meier curves generated with the “survminer” package, with statistical significance assessed via log-rank testing. To investigate potential immune correlates, we performed Spearman correlation analysis between RAD-scores and immune-related genes from the ImmPORT database.

### 2.9 Statistical analyses

For comparative analyses, categorical variables were evaluated using Chi-square or Fisher’s exact test, while continuous variables were analyzed with t-tests (normal distribution) or Mann-Whitney U-tests (non-normal distribution). STAT3 expression thresholds were determined using the minimum *p*-value method. RAD-score cutoffs were established based on imaging histology scores corresponding to the Youden index.

A *p*-value ≤ 0.05 was considered statistically significant. All statistical analyses were performed using SPSS (v26), and R (v4.3.2). Figures were generated with GraphPad Prism (v8.0.0).

## 3 Results

### 3.1 Baseline characteristics of patients

**Figure 2:**
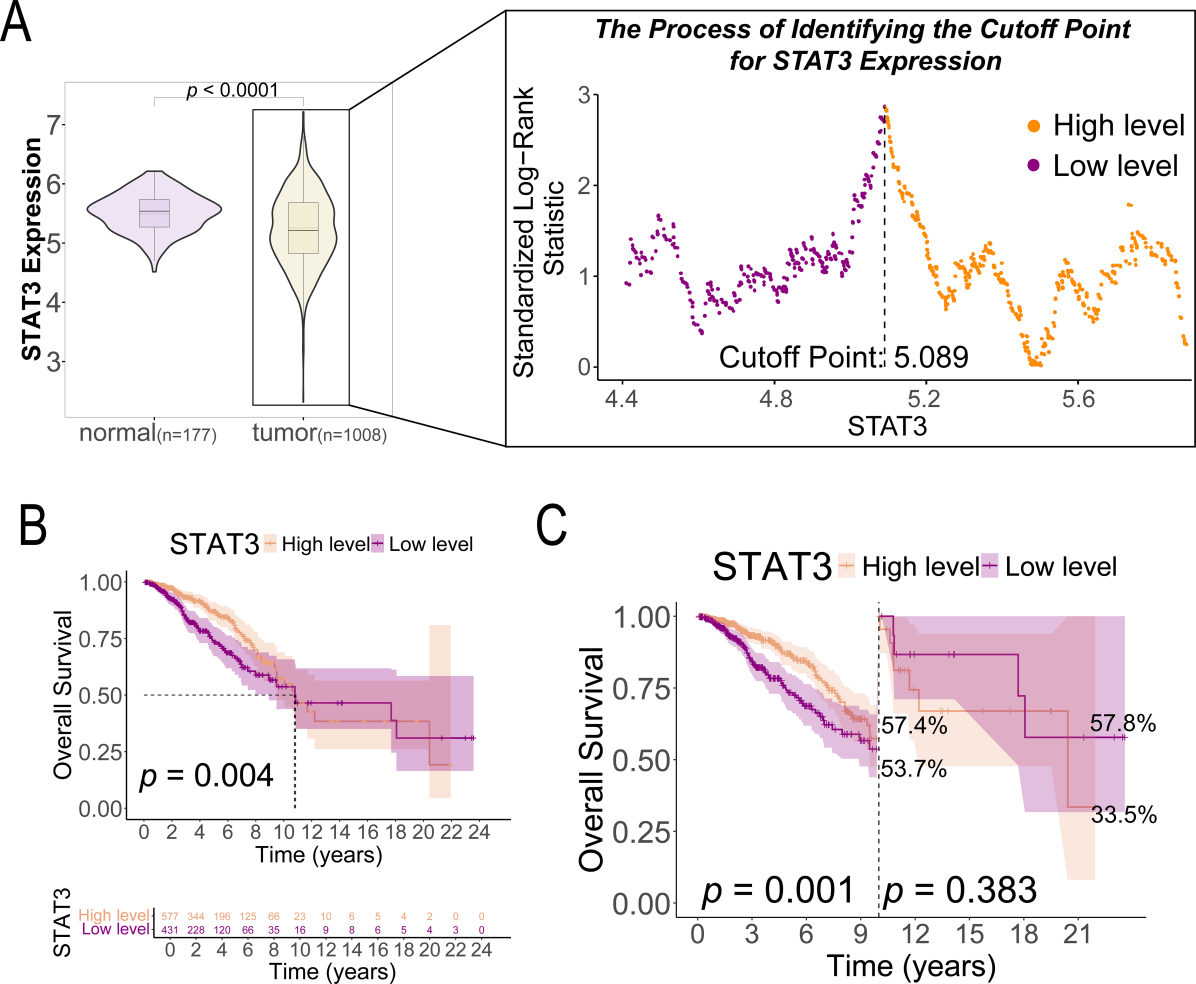
STAT3 gene expression in breast cancer tissues (n = 1008) and normal tissues (n = 177) and its prognostic implications in patients with breast cancer. **(A)** The cutoff value of STAT3 gene expression based on the highest Youden index; **(B)** K-M survival curves comparing OS between high and low STAT3 gene expression groups in patients with breast cancer (n = 1008); **(C)** Landmark analysis of OS between high and low STAT3 gene expression groups in patients with breast cancer (n = 1008).

This study analyzed a cohort of 1,008 breast cancer patients from TCGA along with 80 normal breast tissue samples from GTEx for comparative analysis. We determined the optimal STAT3 expression cutoff value to be 5.089 by using the minimum *p*-value method, which stratified patients into low-expression (n = 431, ≤ 5.089) and high-expression (n = 577, > 5.089) groups. Baseline clinical characteristics are presented in **Table 1**, revealing statistically significant differences between groups for breast carcinoma surgical procedure (*p* = 0.015) and tumor histological type (*p* = 0.007), while other clinical indicators showed no significant variation. Comparative analysis of the combined GTEx and TCGA datasets demonstrated significantly lower STAT3 expression in tumor tissues compared to normal breast specimens (*p* < 0.001, **Figure 2A**).

**Table 1:** Baseline table of STAT3 gene high and low expression groups in breast cancer.

Variables	Total	High expression	Low expression	*P*-value
	(n = 1008)	(n = 577)	(n = 431)	
Age				0.102
< 45	152 (15.1)	75 (13.0)	77 (17.9)	
45 ~ 60	412 (40.9)	241 (41.8)	171 (39.7)	
> 60	444 (44.0)	261 (45.2)	183 (42.4)	
Menopause status				0.886
Premenopausal	217 (21.5)	126 (21.8)	91 (21.1)	
Postmenopausal	647 (64.2)	366 (63.4)	281 (65.2)	
Perimenopausal	35 (3.5)	22 (3.8)	13 (3.0)	
Unknown	109 (10.8)	63 (10.9)	46 (10.7)	
Prior malignancy diagnoses				1
Yes	60 (6.0)	34 (5.9)	26 (6.0)	
No	948 (94.0)	543 (94.1)	405 (94.0)	
Clinical stage at diagnosis				0.158
I/II	742 (73.6)	435 (75.4)	307 (71.2)	
III/IV/Unknown	266 (26.4)	142 (24.6)	124 (28.8)	
Breast carcinoma surgical procedure				0.015
BCS	237 (23.5)	133 (23.1)	104 (24.1)	
Mastectomy	471 (46.7)	252 (43.7)	219 (50.8)	
Unknown	300 (29.8)	192 (33.3)	108 (25.1)	
Axillary lymph node surgical procedure				0.108
Axillary lymph node dissection	533 (52.9)	289 (50.1)	244 (56.6)	
Sentinel node biopsy alone	250 (24.8)	149 (25.8)	101 (23.4)	
Unknown	225 (22.3)	139 (24.1)	86 (20.0)	
Histological type of tumor				0.007
Ductal	710 (70.4)	384 (66.6)	326 (75.6)	
Lobular	192 (19.0)	126 (21.8)	66 (15.3)	
mixed/others	106 (10.5)	67 (11.6)	39 (9.0)	
Pathologic T stage				0.889
T1/T2	845 (83.8)	485 (84.1)	360 (83.5)	
T3/T4/Tx	163 (16.2)	92 (15.9)	71 (16.5)	
Pathologic N stage				0.055
N0/N1/NX	828 (82.1)	486 (84.2)	342 (79.4)	
N2/N3	180 (17.9)	91 (15.8)	89 (20.6)	
Pathologic M stage				0.444
M0	835 (82.8)	483 (83.7)	352 (81.7)	
cM0 (i+)/M1/Mx	173 (17.2)	94 (16.3)	79 (18.3)	
Radiation therapy				0.54
Yes	524 (52.0)	308 (53.4)	216 (50.1)	
No	383 (38.0)	211 (36.6)	172 (39.9)	
Unknown	101 (10.0)	58 (10.1)	43 (10.0)	

BCS, breast-conserving surgery; ALND, axillary lymph node dissection; SNB, sentinel node biopsy.

### 3.2 STAT3 expression levels and ten-year survival

**Figure 3:**
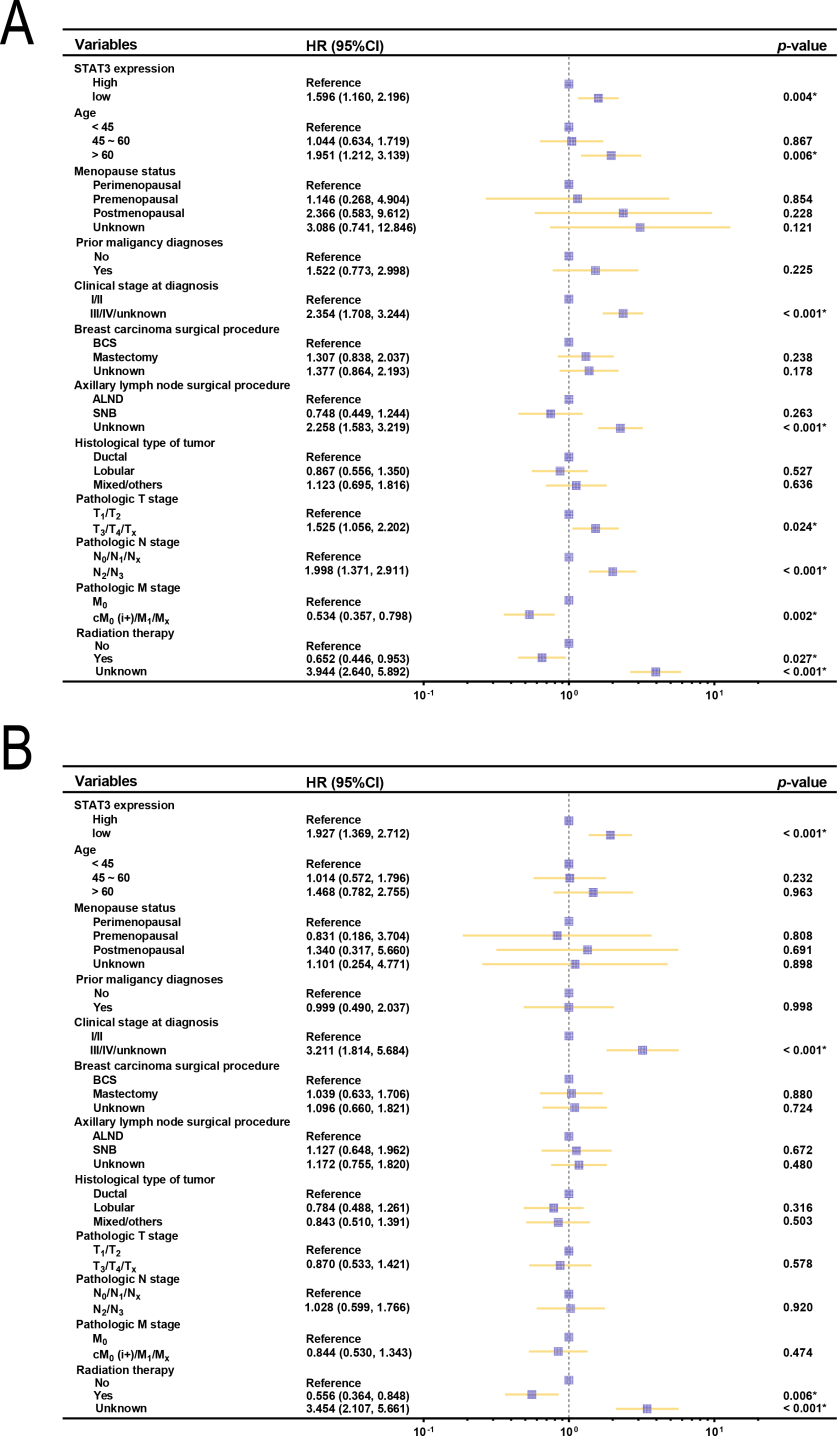
Forest plots of OS and clinical characteristics in patients with breast cancer (n = 1008). **(A)** Forest plot of univariate analysis examining the relationship between OS and clinical characteristics in patients with breast cancer; **(B)** Forest plot of multivariate analysis between OS and clinical characteristics in patients with breast cancer. BCS, breast-conserving surgery; ALND, axillary lymph node dissection; SNB, sentinel node biopsy.

K-M analysis revealed significantly better survival outcomes in the high STAT3 expression group compared to the low-expression cohort (*p* = 0.004, **Figure 2B**). Landmark analysis demonstrated this survival advantage was particularly pronounced within the first 10 years (*p* = 0.001), though the difference attenuated beyond this timeframe (*p* = 0.383, **Figure 2C**). Cox regression analyses confirmed STAT3’s independent prognostic value, with low expression associated with worse overall survival in both univariate (Hazard ratio [HR] = 1.596, 95% CI: 1.160–2.196, *p* = 0.004, **Figure 3A**) and multivariate models (adjusted HR = 1.927, 95% CI: 1.369–2.712, *p* < 0.001, **Figure 3B**). Subgroup analyses identified particularly strong protective associations in non-metastatic patients (HR = 0.525, 95% CI: 0.351–0.786, *p* = 0.002) and those receiving radiotherapy (HR = 0.633, 95% CI: 0.433–0.925, *p* = 0.018) (**Additional file 4**).

### 3.3 GSEA analysis of STAT3 expression and functional pathways

**Figure 4:**
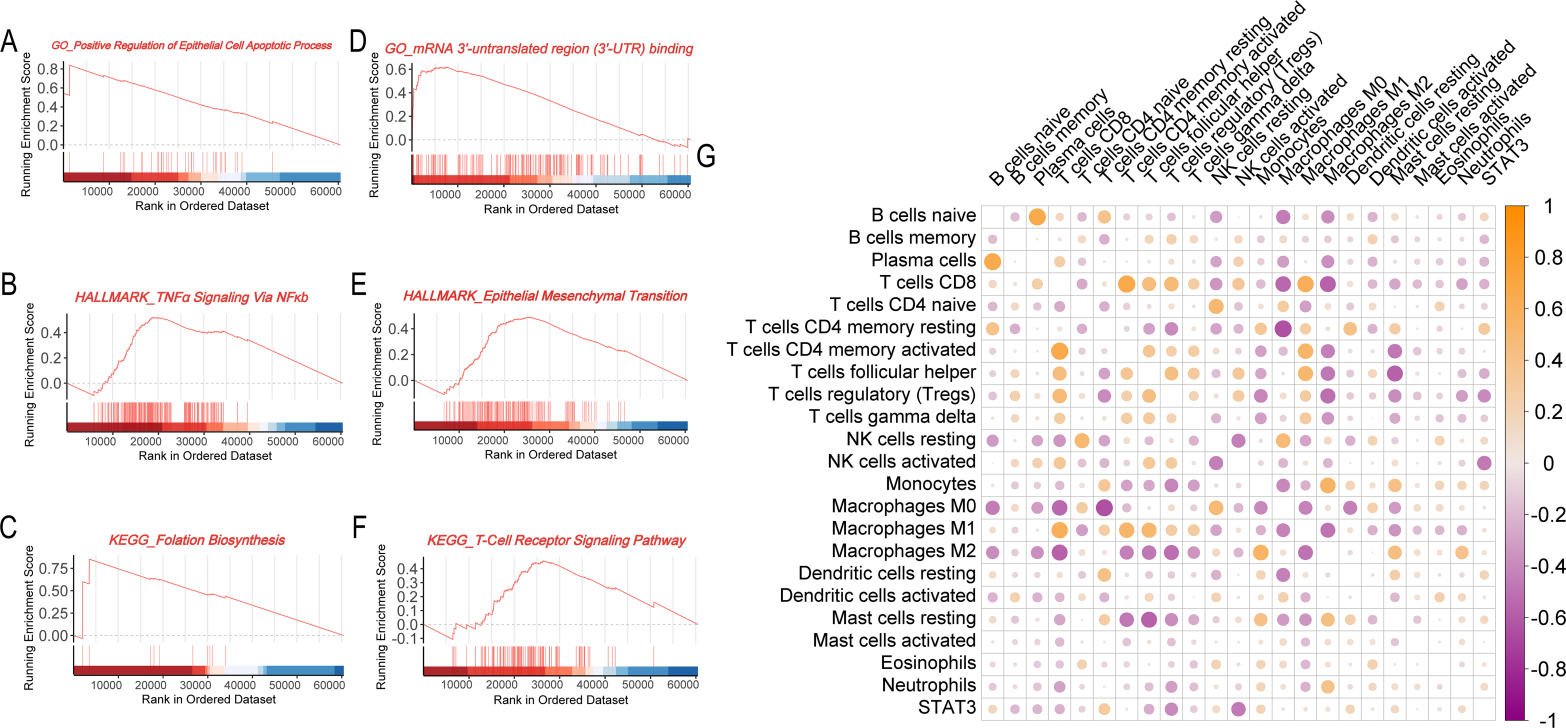
Biological and immunological functions of the STAT3 gene in breast cancer. **(A–C)** GSEA enrichment analysis for the STAT3 high expression group (**A**, GO analysis; **B**, Hallmark pathway analysis; **C**, KEGG pathway analysis); **(D–F)** GSEA enrichment analysis for the STAT3 low expression group (**D**, GO analysis; **E**, Hallmark pathway analysis; **F**, KEGG pathway analysis); **(G)** Correlation matrix showing the relationship between STAT3 gene expression and immune cell infiltration levels in breast cancer tissues.

To elucidate the biological mechanisms underlying the survival advantage observed in patients with high STAT3 expression, we performed GSEA to identify differentially regulated pathways. High STAT3 expression was positively associated with epithelial apoptotic processes (**Figure 4A**), suggesting enhanced tumor suppression through regulated cell death, and with TNF-α signaling via NFκB (**Figure 4B**), indicating robust anti-tumor immunity. The folate biosynthesis pathway (**Figure 4C**) was also enriched, potentially linking metabolic factors to improved prognosis. Conversely, low STAT3 expression correlated with pro-tumorigenic pathways including mRNA 3’-UTR binding (**Figure 4D**) and epithelial-mesenchymal transition (**Figure 4E**), consistent with increased metastatic potential. Notably, T-cell receptor signaling enrichment in low-STAT3 tumors (**Figure 4F**) may reflect T-cell exhaustion, suggesting compromised immune surveillance.

### 3.4 STAT3 expression and immune reaction in patients with breast cancer

Consistent with STAT3’s established immunomodulatory functions (7, 28, 29), we systematically evaluated its association with tumor immune infiltration patterns. Correlation analyses revealed a complex relationship between STAT3 expression and immune cell profiles. Elevated STAT3 levels demonstrated significant negative correlations with cytotoxic immune effectors, including CD8+ T lymphocytes and natural killer (NK) cells, suggesting impairment of antitumor immunity in high-STAT3 tumors. Conversely, STAT3 expression showed positive correlations with immunosuppressive populations, particularly monocytes, M2-polarized macrophages, and tumor-associated neutrophils. Mechanistically, these findings suggest STAT3 promotes an immunosuppressive niche through recruitment of regulatory myeloid cells and subsequent release of immunosuppressive mediators, establishing a self-perpetuating inhibitory microenvironment (**Figure 4G**).

### 3.5 Quantitative analysis of STAT3 protein expression in breast cancer tissues using DIA

**Figure 5:**
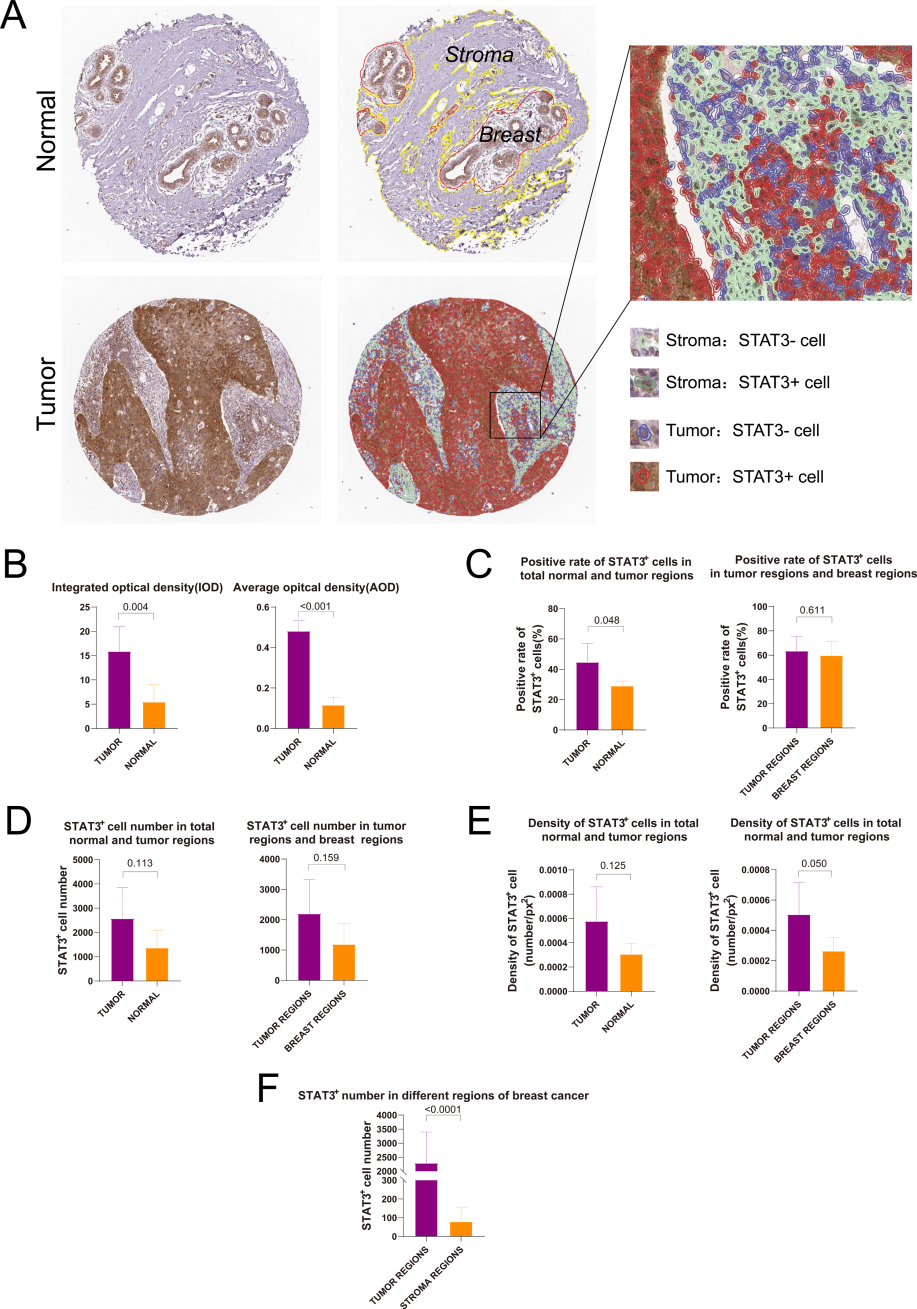
Digital image analysis of immunohistochemical staining images in HPA database. **(A)** Results of tissue segmentation and cell recognition of breast cancer and normal breast tissue; **(B)** Integrated optical density and average optical density of STAT3+ cells in breast cancer and normal breast tissues; **(C)** Rate of STAT3+ cells in breast cancer and normal breast tissues; **(D)** Number of STAT3+ cells in breast cancer and normal breast tissues; **(E)** Density of STAT3+ cells in breast cancer and normal breast tissues; **(F)** Comparison of the number of STAT3+ cells in different regions.

We utilized the DIA software QuPath for both qualitative and quantitative anal-yses of IHC-stained images from the HPA database. By integrating the regions of in-terest delineated by pathologists, facilitated by QuPath, we distinguished between dis-tinct compartments within breast cancer (tumor and stromal regions) and normal breast tissue (breast and stromal components). Furthermore, we identified cells within STAT3-positive (STAT3+) and STAT3-negative (STAT3-) breast cancer subtypes, as well as across various sectors of normal breast tissue (**Figure 5A**). Semi-quantitative anal-ysis demonstrated a statistically significant difference in Integrated Optical Density (IOD) and Average Optical Density (AOD) between breast cancer and normal tissues (IOD: *p* = 0.004; AOD: *p* < 0.001, **Figure 5B**). The positive rate of STAT3+ cells in tumor re-gions was significantly higher compared to region of normal breast tissue (*p* = 0.048, **Figure 5C**). The number and density of STAT3+ cells in tumor tissues were found to be higher compared to those in normal tissues, however, no statistically significant dif-ference was observed (number: *p* = 0.113, **Figure 5D**; density: *p* = 0.125, **Figure 5E**). Notably, in breast cancer tissues, the number of STAT3+ cells within the tumor regions were sig-nificantly greater than that observed in the stromal regions (*p* < 0.0001, **Figure 5F**).

### 3.5 Screening radiomics features and performance evaluation of models

**Figure 6:**
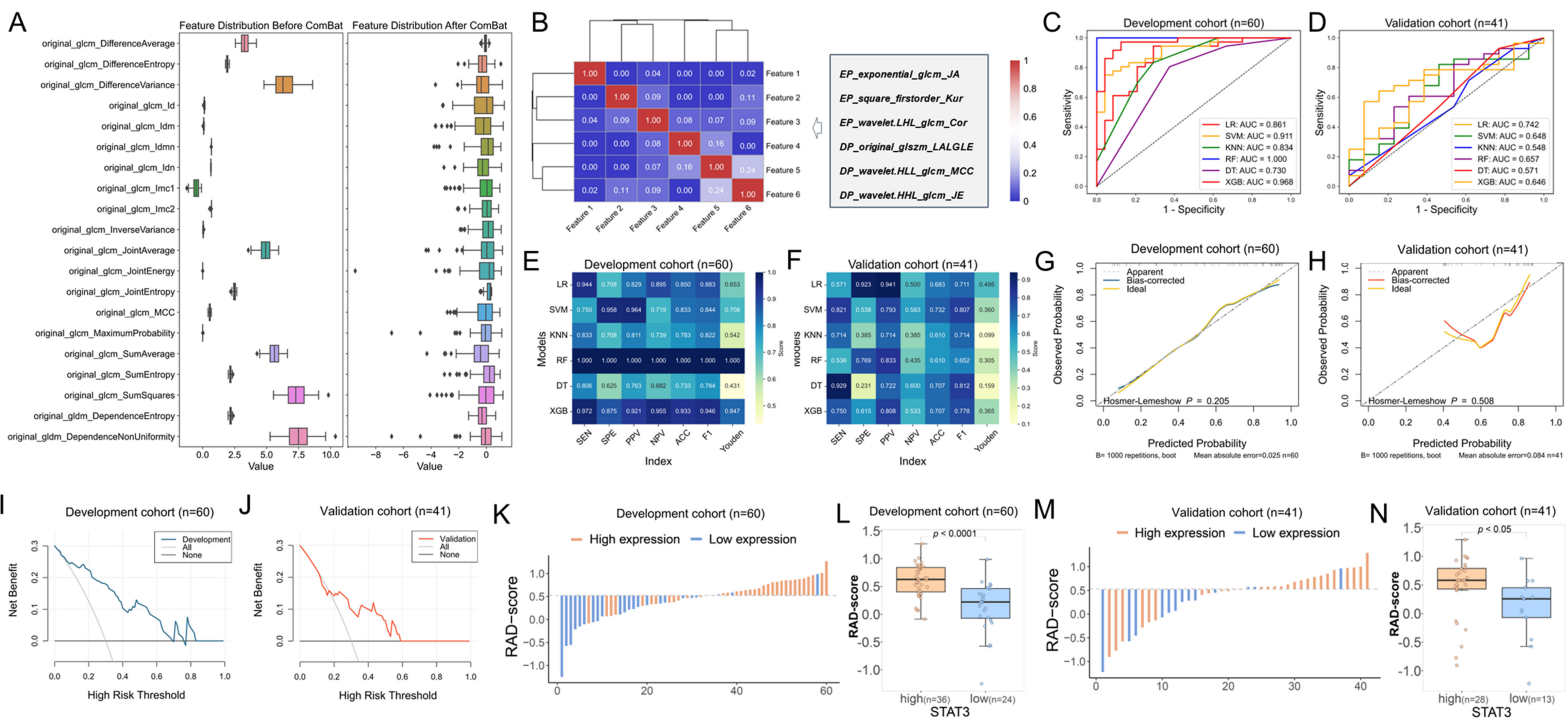
Radiomics model construction and efficacy evaluation results. **(A)** The box diagram of eigenvalue distribution before and after Combat with 20 random features; **(B)** Cluster heatmap of the correlation of selected features; **(C, D)** ROC curves showing the performance of all radiomics models in the development and validation cohorts; **(E, F)** Metric heatmap analysis of diagnostic efficacy parameters for all radiomics models in the development and validation cohorts; **(G, H)** Calibration curves depicting the agreement between predicted and observed outcomes based on LR model in the development and validation cohorts; **(I, J)** DCA curves evaluating the clinical utility of the LR model in the development and validation cohorts; **(K)** Waterfall plot illustrating the distribution of radiomics scores (RAD-scores) in the development cohort; **(L)** Box plot showing the differences in distribution of RAD-scores between high and low STAT3 expression groups in the development cohort; **(M)** Waterfall plot of RAD-scores in the validation cohort; **(N)** Box plot comparing RAD-score distributions between high and low STAT3 expression groups in the validation cohort.

The study cohort comprised 101 patients, randomly divided into development (n = 60) and validation (n = 41) sets at a 6:4 ratio, with balanced clinical characteristics and STAT3 expression between groups (*p* > 0.05, **Additional file 5**). From multiphase MRI, 3,100 radiomic features were initially extracted. Following combat analysis (**Figure 6A**), quality control (ICC ≥ 0.70, **Additional file 6**), Pearson correlation analysis, and LASSO regression with 5-fold cross-validation (optimal λ = 0.081), six robust features (three each from early- and delayed-phase images) were identified at last (Figure B). The weight coefficients of each selected features inherently represent their importance were shown in **Additional file 7**, and the clinical and biological interpretations were detailed in **Additional file 8**.

The analysis revealed that while the RF model showed significantly higher AUC than LR in the training cohort (*p* = 0.029), other models demonstrated only marginal improvements over LR (**Additional file 9**). Notably, both LR and DT models maintained consistent performance across development and validation cohorts (*p* > 0.05), indicating acceptable generalizability, whereas other models exhibited varying degrees of overfitting. When comparing the two stable models (**Additional file 9**), LR demonstrated superior discriminative ability in both cohorts (development: 0.861, 95% CI [0.749 - 0.947], **Figure 6C**; validation: 0.742, 95% CI [0.588 - 0.884], **Figure 6D**, p = 0.209) compared to DT (development: 0.730, 95% CI [0.615 - 0.837], **Figure 6C**; validation: 0.571, 95% CI [0.376 - 0.746], **Figure 6D**, p = 0.089). Based on this robust performance and stability across datasets, we selected the LR model as the optimal predictive model for our study. The performance matrix analysis (**Figures 6E, F**) further confirmed that the LR model achieved the highest specificity (92.3%), PPV (94.1%), and Youden index (0.495) in the validation cohort. Bootstrap validation confirmed LR model stability (mean AUC = 0.822, 95%CI: 0.780–0.847, **Additional file 10**). Calibration analysis showed excellent fit in both cohorts (*p* = 0.412 development, *p* = 0.088 validation; **Figures 6G, H**). DCA demonstrated clinical utility across probability thresholds of 0%-70% (development, **Figure 6I**) and 20% - 50% (validation, **Figure 6J**). These results validate the LR model’s accuracy, reliability, and clinical applicability for breast cancer stratification.

The Rad-score was calculated using the LR-based model and compared between STAT3-low and STAT3-high expression groups in both the development and validation cohorts. Significant RAD-score differences were observed between STAT3 expression groups in both development (median [IQR]: 0.222 [-0.070, 0.465] vs 0.628 [0.405, 0.844], *p* < 0.001, **Figures 6K–L**) and validation cohorts (0.258 [-0.069, 0.452] vs 0.582 [0.430, 0.789], *p* = 0.018, **Figures 6M, N**).

### 3.6 Relationship between RAD-scores and patient survival

**Figure 7:**
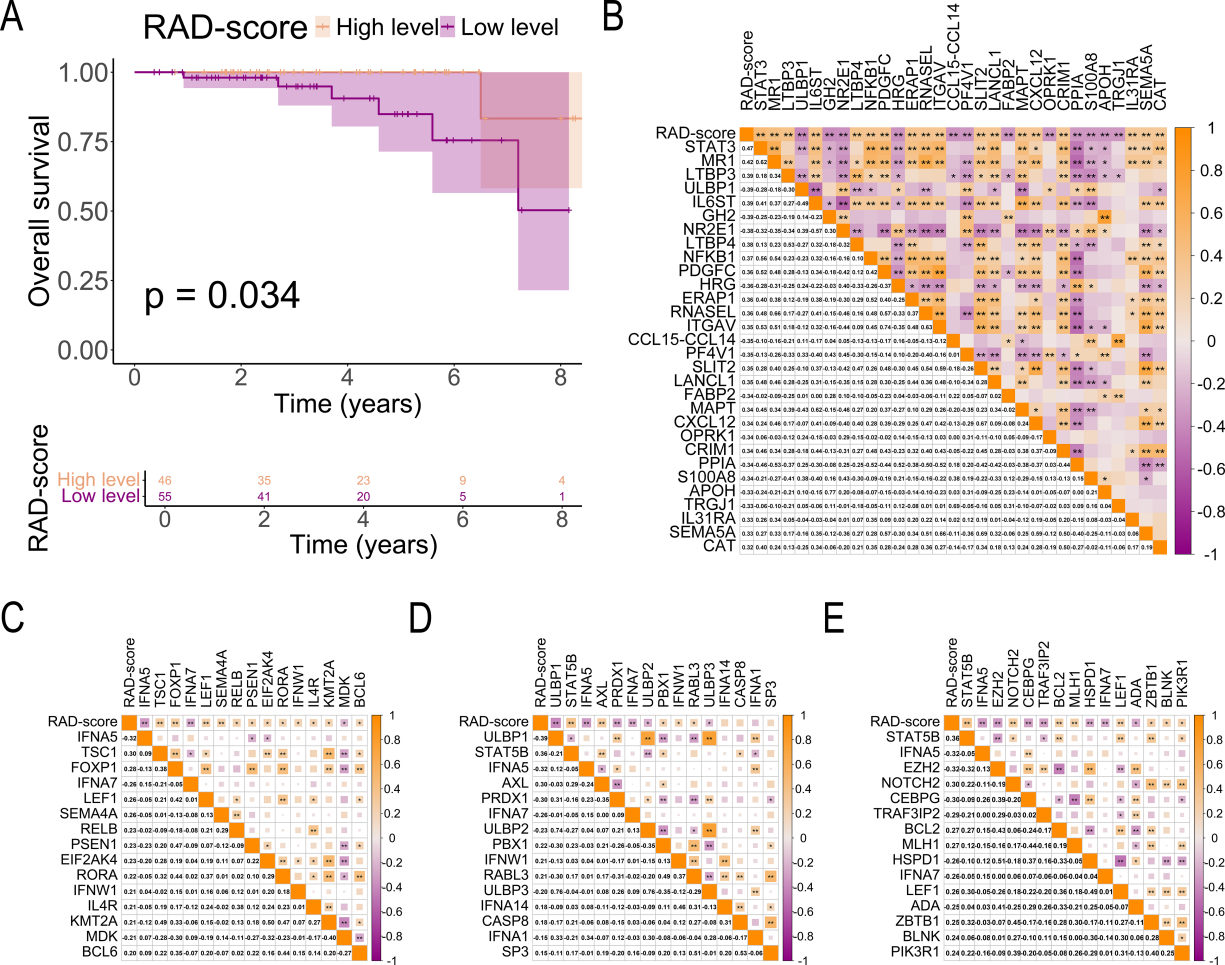
Association of RAD-score With Prognosis and Immune-Related Genes. **(A)** K-M curves comparing survival outcomes between high and low RAD-score groups in patients with breast cancer; **(B)** Correlation matrix illustrating the relationship between RAD-scores and immune cell-related gene expression in breast cancer tissues; **(C–E)** Correlation matrix between RAD-score and immune cell-related gene expression in breast cancer tissues (**C**, T cell-related genes; **D**, NK cell-related genes; **E**, B cell-related genes). The symbols "*" and "**" indicate statistical significance levels of p < 0.05 and p < 0.01, respectively.

The prognostic value of RAD-scores was evaluated by analyzing their association with OS in the 101-patient cohort. Using the Youden index-derived cutoff (0.523), patients were stratified into high (n = 47) and low (n = 54) RAD-score groups. K-M analysis demonstrated significantly improved OS in the high-RAD group (*p* = 0.034, **Figure 7A**), confirming the model’s prognostic capability.

### 3.7 Relationship between RAD-scores and tumor immune-related genes

Our analysis revealed significant immunogenomic correlations with RAD-scores in breast cancer. A strong positive association was observed between RAD-scores and STAT3 expression (r = 0.47, *p* < 0.01, **Figure 7B**), with similar correlation patterns for immune-related genes. Specifically, RAD-scores showed significant positive correlations with:

- T-cell related genes (TSC1, FOXP1, LEF1, SEMA4A, RELB, PSEN1, EIF2AK4, RORA, IFNW1, IL4R, KMT2A, BCL6; *p* < 0.05, **Figure 7C**)
- NK-cell markers (STAT5B, AXL, PBX1, IFNW1, RABL3; *p* < 0.05, **Figure 7D**)
- B-cell associated genes (NOTCH2, BCL2, MLH1, LEF1, ZBTB1, BLNK, PIK3R1; *p* < 0.05, **Figure 7E**)

## 4 Discussion

Breast cancer biomarker research remains in nascent stages, with no single prognostic marker achieving universal clinical adoption. Among emerging candidates, STAT3 has emerged as a critical regulator in breast cancer progression, particularly in triple-negative subtypes, demonstrating strong prognostic value and immunotherapy response associations (10, 30). These characteristics position STAT3 expression status as a crucial determinant for personalized treatment strategies.

Our study findings demonstrate the complex role of STAT3 in breast cancer, particularly its complex relationship with immune regulation and patient prognosis, which aligns with existing literature that emphasizes the importance of STAT3 as a prognostic factor and potential biomarker for immunotherapy responses in breast cancer (31, 32), and this phenomenon may contribute to the diminished ten-year survival rates observed in these patients, reflecting the complex interplay between STAT3 expression patterns and immune activity Additionally, our study developed a novel radiomics approach using DCE-MRI to non-invasively predict STAT3 expression, bridging imaging and genomic analysis in precision oncology.

Our study revealed higher STAT3 mRNA levels in normal tissues compared to breast cancer tissues, challenging the conventional oncogenic view of STAT3 (33). This paradox underscores the complexity of its role in cancer biology, necessitating evaluation of multiple factors, including mRNA/protein expression, activation states (e.g., phosphorylation), and context-dependent signaling pathway interactions. Notably, STAT3 expression correlated significantly with immune modulation, particularly through suppressed NK cell activity—a key anti-tumor mechanism—potentially facilitating immune evasion via impaired IFN-γ and TNF-α production (34, 35). While our CIBERSORTx analysis identified these immune correlates, the computational nature of deconvolution warrants validation through flow cytometry or spatial transcriptomics in future studies.

The GSEA analysis revealed enrichment of immune-suppressive pathways and exhaustion markers in low-STAT3 tumors, suggesting STAT3 deficiency may drive immune evasion through both immunosuppressive cell recruitment and T-cell dysfunction. This aligns with evidence that STAT3 loss promotes TGF-β-mediated suppression and PD-L1 upregulation, while impairing T-cell metabolic fitness through oxidative stress (36, 37). The combined effects of tumor cell p-STAT3 hyperactivation and STAT3 mRNA deficiency likely create a dual immunosuppressive axis, though future studies should dissect cell-type-specific effects using spatial transcriptomics.

The prognostic paradox of low STAT3 mRNA associating with poorer survival may reflect compensatory hyperactivation through post-transcriptional modifications. Growing evidence suggests STAT3’s functional activity is primarily regulated through phosphorylation status rather than mRNA abundance (38). While moderate STAT3 activity maintains normal T-cell function (39), its deficiency may drive T-cell exhaustion, evidenced by enriched TCR signaling pathways as a compensatory mechanism. This aligns with studies demonstrating that STAT3 activation (p-STAT3), not total STAT3 mRNA levels, drives tumor progression and poor survival, likely via cell-autonomous and immune-mediated mechanisms (40). Although our study identifies a correlation between low STAT3 expression and T cell exhaustion, the downstream mechanisms remain unclear. Prior work suggests STAT3 may regulate key exhaustion-related genes, such as PD-1 and TOX (41), and modulate T cell metabolism (42). While our data do not functionally validate these targets, the observed exhaustion signature aligns with potential STAT3-dependent regulation. Further studies could clarify these mechanistic links.

Contrary to previous reports of STAT3 upregulation in breast cancer (11, 12), our integrated TCGA/GTEx analysis demonstrated higher constitutive expression in normal tissues, with survival analysis showing no significant prognostic impact of mRNA levels alone. This dichotomy suggests STAT3’s physiological role in normal tissue homeostasis (43) versus its cancer-specific hyperactivation through post-transcriptional mechanisms (44). Critically, tumor progression appears driven not by transcriptional overexpression but by dysregulated activation states, particularly phosphorylation-mediated signaling (45, 46). These findings redefine STAT3’s oncogenic paradigm, emphasizing that malignant progression depends more on post-transcriptional activation than mRNA abundance, with important implications for targeted therapeutic strategies.

Recent advances in medical imaging and computer science have established radiomics as a powerful tool for breast cancer research (47). Multisequence, multiparametric breast MRI enables extraction of high-throughput radiomic features that provide novel biological insights, predict disease progression, and guide personalized treatment strategies. The emerging field of radiomics has demonstrated particular promise by correlating multimodal imaging data (MRI/CT) with genomic profiles to improve prognostic predictions (48–50). This approach has revealed significant associations between imaging features (tumor morphology, texture) and molecular characteristics, including gene expression patterns in the tumor microenvironment (51, 52). Given the established association between high STAT3 expression and poor prognosis (53), we hypothesized that integrating STAT3 expression profiles with advanced radiomic analysis could enhance prognostic accuracy.

In this study, we developed a novel MRI-based radiomics model to predict prognosis and immunotherapeutic characteristics based on STAT3 expression. Leveraging MRI’s superior soft-tissue resolution and multiphasic contrast enhancement capabilities, so the signal intensity and texture features of these images correlate with tumor heterogeneity and may also serve as predictors of its biological behavior (54). We identified six statistically significant radiomic features associated with STAT3 expression: one first-order feature (Square_Firstorder_Kurtosis), two texture features, and three wavelet-transformed higher-order features. Notably, the two most predictive features (regression coefficients > 10) derived from delayed-phase images included: (1) wavelet.HHL_glcm_JointEnergy (Coef = 122.452), reflecting tumor texture uniformity, and (2) original_glszm_LargeAreaLowGrayLevelEmphasis (Coef = 32.437), characterizing tissue distribution patterns (55). Through comprehensive evaluation of multiple machine learning classifiers, the LR-based model demonstrated superior generalizability in the validation cohort compared to alternative approaches. Despite comparable AUC to other models, LR was selected for its lower performance variance and higher specificity—a clinical priority to minimize costly false positives in molecular profiling. This finding supports the selection of the LR model as a robust, non-invasive tool for characterizing STAT3 expression levels, offering potential clinical utility for tumor phenotyping.

Our study advances previous research by uniquely integrating STAT3 expression analysis with advanced radiomics, revealing new relationships between molecular processes and imaging phenotypes. The identified features not only capture subvisual tumor heterogeneity but also demonstrate significant associations with gene expression and survival outcomes. Future directions include multi-omics integration for enhanced predictive modeling, prospective clinical validation, and investigation of radiomic-immune microenvironment correlations. While clinical translation requires further validation, our findings demonstrate radiomics’ potential as a non-invasive tool for STAT3 expression profiling and prognostic assessment in breast cancer, representing a significant step toward precision oncology.

However, several limitations should be acknowledged in this study. First, this study focused on OS due to data availability constraints in public repositories. Future work should integrate disease-free survival and treatment-response metrics through prospective collaborations with clinical centers. Second, potential biases may exist as all DCE-MRI data were obtained from public repositories, and although ComBat harmonization was applied, residual variability across scanners may persist. Third, the smaller sample size (particularly in the TCIA cohort) may limit generalizability, warranting external validation in larger multicenter cohorts - an effort we are actively pursuing through expanded collaborations. Lastly, incomplete immunohistochemical data precluded molecular subtype analyses, potentially masking subtype-specific radiomic-STAT3 relationships. These limitations highlight the need for prospective multicenter studies with standardized protocols.

## 5 Conclusions

This study establishes STAT3 as a key prognostic biomarker in breast cancer and demonstrates the clinical potential of our validated DCE-MRI radiomics model for noninvasive STAT3 assessment. These findings advance precision oncology by enabling imaging-based prediction of tumor biology and treatment response, supporting personalized therapeutic strategies.

## Data Availability

The data used in this study are openly accessible from various public sources, including the UCSC Xena Database (https://xena.ucsc.edu/), The Cancer Imaging Archive (TCIA) for the TCGA-BRCA collection (https://www.cancerimagingarchive.net/collection/tcga-brca/), The Human Protein At-las (HPA) (https://www.proteinatlas.org/), ImmPort Shared Data (https://www.immport.org/shared/home), and the GTEx Database (https://www.gtexportal.org/).

## Supplementary material

Additional file 1 Box Plot Illustrating the Removal of Batch Effects from STAT3 Gene Expression Data in the TCGA and GTEx Databases. (File format:.tif).

Additional file 2 Gene List Retrieved from the ImmPORT Database. (File format:.xlsx).

Additional file 3 Hyperparameter optimization during machine learning models construction. (File format:.docx).

Additional file 4 Forest Plot of Subgroup Cox Regression Analysis. (File format:.tif).

Additional file 5 Comparison of the Characteristics of the Development and Validation Cohorts. (File format:.docx).

Additional file 6 ICC Values for Radiomics Features. (File format:.xlsx).

Additional file 7 Weighted Coef Values of Each Features. (File format:.docx).

Additional file 8 Description of selected radiomic features and their clinical relevance. (File format:.docx).

Additional file 9 DeLong’s test between models within and across development validation cohorts. (File format:.tif).

Additional file 10 ROC Curve Derived from Bootstrap Analysis. (File format:.tif).

## References

[1] Sung H Ferlay J Siegel RL Laversanne M Soerjomataram I Jemal A. Global cancer statistics 2020: GLOBOCAN estimates of incidence and mortality worldwide for 36 cancers in 185 countries. CA Cancer J Clin. (2021) 71:209–49. doi: 10.3322/caac.21660 33538338

[2] Loibl S André F Bachelot T Barrios CH Bergh J Burstein HJ. Early breast cancer: ESMO Clinical Practice Guideline for diagnosis, treatment and follow-up. Ann Oncol. (2024) 35:159–82. doi: 10.1016/j.annonc.2023.11.016 38101773

[3] Goldhirsch A Winer EP Coates AS Gelber RD Piccart-Gebhart M Thürlimann B. Personalizing the treatment of women with early breast cancer: highlights of the St Gallen International Expert Consensus on the Primary Therapy of Early Breast Cancer 2013. Ann Oncol. (2013) 24:2206–23. doi: 10.1093/annonc/mdt303 PMC3755334 23917950

[4] Li J Chen Z Su K Zeng J. Clinicopathological classification and traditional prognostic indicators of breast cancer. Int J Clin Exp Pathol. (2015) 8:8500–5. PMC4555752 26339424

[5] Pescia C Guerini-Rocco E Viale G Fusco N. Advances in early breast cancer risk profiling: from histopathology to molecular technologies. Cancers. (2023) 15:5430. doi: 10.3390/cancers15225430 38001690 PMC10670146

[6] Vellan CJ Islam T De Silva S Mohd Taib NA Prasanna G Jayapalan JJ. Exploring novel protein-based biomarkers for advancing breast cancer diagnosis: A review. Clin Biochem. (2024) 129:110776. doi: 10.1016/j.clinbiochem.2024.110776 38823558

[7] Zou S Tong Q Liu B Huang W Tian Y Fu X. Targeting STAT3 in cancer immunotherapy. Mol Cancer. (2020) 19:145. doi: 10.1186/s12943-020-01258-7 32972405 PMC7513516

[8] Santoni M Miccini F Cimadamore A Piva F Massari F Cheng L. An update on investigational therapies that target STAT3 for the treatment of cancer. Expert Opin investigational Drugs. (2021) 30:245–51. doi: 10.1080/13543784.2021.1891222 33599169

[9] Li T Guo H Zhao X Jin J Zhang L Li H. Gastric cancer cell proliferation and survival is enabled by a cyclophilin B/STAT3/miR-520d-5p signaling feedback loop. Cancer Res. (2017) 77:1227–40. doi: 10.1158/0008-5472.Can-16-0357 28011625

[10] van Pul KM Vuylsteke R de Beijer MTA van de Ven R van den Tol MP Stockmann H. Breast cancer-induced immune suppression in the sentinel lymph node is effectively countered by CpG-B in conjunction with inhibition of the JAK2/STAT3 pathway. J immunotherapy Cancer. (2020) 8:e000761. doi: 10.1136/jitc-2020-000761 PMC7552844 33046620

[11] Lin WH Dai WG Xu XD Yu QH Zhang B Li J. Downregulation of DPF3 promotes the proliferation and motility of breast cancer cells through activating JAK2/STAT3 signaling. Biochem Biophys Res Commun. (2019) 514:639–44. doi: 10.1016/j.bbrc.2019.04.170 31076105

[12] Chang R Song L Xu Y Wu Y Dai C Wang X. Loss of Wwox drives metastasis in triple-negative breast cancer by JAK2/STAT3 axis. Nat Commun. (2018) 9:3486. doi: 10.1038/s41467-018-05852-8 30154439 PMC6113304

[13] Ma Q Gao FF He X Li K Gao Y Xu XL. Antitumor effects of saikosaponin b2 on breast cancer cell proliferation and migration. Mol Med Rep. (2019) 20:1943–51. doi: 10.3892/mmr.2019.10385 31257464

[14] Lee H Jeong AJ Ye SK. Highlighted STAT3 as a potential drug target for cancer therapy. BMB Rep. (2019) 52:415–23. doi: 10.5483/BMBRep.2019.52.7.152 PMC6675244 31186087

[15] Liu Z Duan T Zhang Y Weng S Xu H Ren Y. Radiogenomics: a key component of precision cancer medicine. Br J Cancer. (2023) 129:741–53. doi: 10.1038/s41416-023-02317-8 PMC10449908 37414827

[16] Zhao W Yang J Ni B Bi D Sun Y Xu M. Toward automatic prediction of EGFR mutation status in pulmonary adenocarcinoma with 3D deep learning. Cancer Med. (2019) 8:3532–43. doi: 10.1002/cam4.2233 PMC6601587 31074592

[17] Taouli B Hoshida Y Kakite S Chen X Tan PS Sun X. Imaging-based surrogate markers of transcriptome subclasses and signatures in hepatocellular carcinoma: preliminary results. Eur Radiol. (2017) 27:4472–81. doi: 10.1007/s00330-017-4844-6 PMC5654702 28439654

[18] Xie Y Wang M Xia H Sun H Yuan Y Jia J. Development and validation of a CECT-based radiomics model for predicting IL1B expression and prognosis of head and neck squamous cell carcinoma. Front Oncol. (2023) 13:1121485. doi: 10.3389/fonc.2023.1121485 36969073 PMC10036854

[19] Hu LS Ning S Eschbacher JM Gaw N Dueck AC Smith KA. Multi-parametric MRI and texture analysis to visualize spatial histologic heterogeneity and tumor extent in glioblastoma. PloS One. (2015) 10:0141506. doi: 10.1371/journal.pone.0141506 PMC4658019 26599106

[20] Mao L Chen H Liang M Li K Gao J Qin P. Quantitative radiomic model for predicting Malignancy of small solid pulmonary nodules detected by low-dose CT screening. Quantitative Imaging Med Surg. (2019) 9:263–72. doi: 10.21037/qims.2019.02.02 PMC6414768 30976550

[21] Pandit H Hong YK Li Y Rostas J Pulliam Z Li SP. Evaluating the regulatory immunomodulation effect of irreversible electroporation (IRE) in pancreatic adenocarcinoma. Ann Surg Oncol. (2019) 26:800–6. doi: 10.1245/s10434-018-07144-3 30610562

[22] Weinstein JN Collisson EA Mills GB Shaw KR Ozenberger BA Ellrott K. The Cancer Genome Atlas Pan-Cancer analysis project. Nat Genet. (2013) 45:1113–20. doi: 10.1038/ng.2764 PMC3919969 24071849

[23] GTEx Consortium. The genotype-tissue expression (GTEx) project. Nat Genet. (2013) 45:580–5. doi: 10.1038/ng.2653 PMC4010069 23715323

[24] Lingle W Erickson BJ Zuley ML Jarosz R Bonaccio E Filippini J. The cancer genome atlas breast invasive carcinoma collection (TCGA-BRCA) (Version 3). The Cancer Imaging Archive (2016). doi: 10.7937/K9/TCIA.2016.AB2NAZRP

[25] Goldman MJ Craft B Hastie M Repečka K McDade F Kamath A. Visualizing and interpreting cancer genomics data via the Xena platform. Nat Biotechnol. (2020) 38:675–8. doi: 10.1038/s41587-020-0546-8 PMC7386072 32444850

[26] Bhattacharya S Andorf S Gomes L Dunn P Schaefer H Pontius J. ImmPort: disseminating data to the public for the future of immunology. Immunologic Res. (2014) 58:234–9. doi: 10.1007/s12026-014-8516-1 24791905

[27] Newman AM Steen CB Liu CL Gentles AJ Chaudhuri AA Scherer F. Determining cell type abundance and expression from bulk tissues with digital cytometry. Nat Biotechnol. (2019) 37:773–82. doi: 10.1038/s41587-019-0114-2 PMC6610714 31061481

[28] Ma JH Qin L Li X. Role of STAT3 signaling pathway in breast cancer. Cell communication signaling: CCS. (2020) 18:33. doi: 10.1186/s12964-020-0527-z 32111215 PMC7048131

[29] Siveen KS Sikka S Surana R Dai X Zhang J Kumar AP. Targeting the STAT3 signaling pathway in cancer: role of synthetic and natural inhibitors. Biochim Biophys Acta. (2014) 1845:136–54. doi: 10.1016/j.bbcan.2013.12.005 24388873

[30] Taifour T Attalla SS Zuo D Gu Y Sanguin-Gendreau V Proud H. The tumor-derived cytokine Chi3l1 induces neutrophil extracellular traps that promote T cell exclusion in triple-negative breast cancer. Immunity. (2023) 56:2755–2772.e2758. doi: 10.1016/j.immuni.2023.11.002 38039967

[31] Zhou J Wan F Wang L Peng C Huang R Peng F. STAT4 facilitates PD-L1 level via IL-12R/JAK2/STAT3 axis and predicts immunotherapy response in breast cancer. MedComm. (2023) 4:e464. doi: 10.1002/mco2.464 38107057 PMC10724500

[32] Chuangchot N Jamjuntra P Yangngam S Luangwattananun P Thongchot S Junking M. Enhancement of PD-L1-attenuated CAR-T cell function through breast cancer-associated fibroblasts-derived IL-6 signaling via STAT3/AKT pathways. Breast Cancer research: BCR. (2023) 25:86. doi: 10.1186/s13058-023-01684-7 37480115 PMC10362675

[33] Kaminskiy Y Melenhorst JJ. STAT3 role in T-cell memory formation. Int J Mol Sci. (2022) 23:2878. doi: 10.3390/ijms23052878 35270020 PMC8910982

[34] Thanapati S Ganu M Giri P Kulkarni S Sharma M Babar P. Impaired NK cell functionality and increased TNF-α production as biomarkers of chronic chikungunya arthritis and rheumatoid arthritis. Hum Immunol. (2017) 78:370–4. doi: 10.1016/j.humimm.2017.02.006 28213049

[35] Wang R Jaw JJ Stutzman NC Zou Z Sun PD. Natural killer cell-produced IFN-γ and TNF-α induce target cell cytolysis through up-regulation of ICAM-1. J leukocyte Biol. (2012) 91:299–309. doi: 10.1189/jlb.0611308 22045868 PMC3290424

[36] Bai R Hao L Zhou G Fu Q Zhang P Lin P. The mechanism of TGF-β mediating BRD4/STAT3 signaling pathway to promote fibroblast proliferation and thus promote keloid progression. Heliyon. (2024) 10:e38188. doi: 10.1016/j.heliyon.2024.e38188 39391472 PMC11466596

[37] Xie C Zhou X Liang C Li X Ge M Chen Y. Apatinib triggers autophagic and apoptotic cell death via VEGFR2/STAT3/PD-L1 and ROS/Nrf2/p62 signaling in lung cancer. J Exp Clin Cancer research: CR. (2021) 40:266. doi: 10.1186/s13046-021-02069-4 34429133 PMC8385858

[38] Ishteyaque S Singh G Yadav KS Verma S Sharma RK Sen S. Cooperative STAT3-NFkB signaling modulates mitochondrial dysfunction and metabolic profiling in hepatocellular carcinoma. Metabolism. (2024) 152:155771. doi: 10.1016/j.metabol.2023.155771 38184165

[39] McIlwain DR Grusdat M Pozdeev VI Xu HC Shinde P Reardon C. T-cell STAT3 is required for the maintenance of humoral immunity to LCMV. Eur J Immunol. (2015) 45:418–27. doi: 10.1002/eji.201445060 PMC4383653 25393615

[40] Pan YM Wang CG Zhu M Xing R Cui JT Li WM. STAT3 signaling drives EZH2 transcriptional activation and mediates poor prognosis in gastric cancer. Mol cancer. (2016) 15:79. doi: 10.1186/s12943-016-0561-z 27938379 PMC5148878

[41] Cao C Xu M Wei Y Peng T Lin S Liu X. CXCR4 orchestrates the TOX-programmed exhausted phenotype of CD8(+) T cells via JAK2/STAT3 pathway. Cell Genomics. (2024) 4:100659. doi: 10.1016/j.xgen.2024.100659 39317187 PMC11602566

[42] Sun Q Zhao X Li R Liu D Pan B Xie B. STAT3 regulates CD8+ T cell differentiation and functions in cancer and acute infection. J Exp Med. (2023) 220:e20220686. doi: 10.1084/jem.20220686 36688918 PMC9884582

[43] Clarkson RW Boland MP Kritikou EA Lee JM Freeman TC Tiffen PG. The genes induced by signal transducer and activators of transcription (STAT)3 and STAT5 in mammary epithelial cells define the roles of these STATs in mammary development. Mol Endocrinol (Baltimore Md.). (2006) 20:675–85. doi: 10.1210/me.2005-0392 16293640

[44] Hughes K Watson CJ. The multifaceted role of STAT3 in mammary gland involution and breast cancer. Int J Mol Sci. (2018) 19:1695. doi: 10.3390/ijms19061695 29875329 PMC6032292

[45] Zhao Y Hu Z Li J Hu T. EZH2 exacerbates breast cancer by methylating and activating STAT3 directly. J Cancer. (2021) 12:5220–30. doi: 10.7150/jca.50675 PMC8317538 34335938

[46] Rébé C Ghiringhelli F. STAT3, a master regulator of anti-tumor immune response. Cancers. (2019) 11:1280. doi: 10.3390/cancers11091280 31480382 PMC6770459

[47] Conti A Duggento A Indovina I Guerrisi M Toschi N. Radiomics in breast cancer classification and prediction. Semin Cancer Biol. (2021) 72:238–50. doi: 10.1016/j.semcancer.2020.04.002 32371013

[48] Tagliafico AS Piana M Schenone D Lai R Massone AM Houssami N. Overview of radiomics in breast cancer diagnosis and prognostication. Breast (Edinburgh Scotland). (2020) 49:74–80. doi: 10.1016/j.breast.2019.10.018 31739125 PMC7375670

[49] Zheng X Yao Z Huang Y Yu Y Wang Y Liu Y. Deep learning radiomics can predict axillary lymph node status in early-stage breast cancer. Nat Commun. (2020) 11:1236. doi: 10.1038/s41467-020-15027-z 32144248 PMC7060275

[50] LoGullo R Horvat J Reiner J Pinker K. Multimodal, multiparametric and genetic breast imaging. Der Radiologe. (2021) 61:183–91. doi: 10.1007/s00117-020-00801-3 33464404

[51] Fan M Wang K Zhang Y Ge Y Lü Z Li L. Radiogenomic analysis of cellular tumor-stroma heterogeneity as a prognostic predictor in breast cancer. J Trans Med. (2023) 21:851. doi: 10.1186/s12967-023-04748-6 PMC10675940 38007511

[52] Su GH Xiao Y You C Zheng RC Zhao S Sun SY. Radiogenomic-based multiomic analysis reveals imaging intratumor heterogeneity phenotypes and therapeutic targets. Sci Adv. (2023) 9:eadf0837. doi: 10.1126/sciadv.adf0837 37801493 PMC10558123

[53] Aleskandarany MA Agarwal D Negm OH Ball G Elmouna A Ashankyty I. The prognostic significance of STAT3 in invasive breast cancer: analysis of protein and mRNA expressions in large cohorts. Breast Cancer Res Treat. (2016) 156:9–20. doi: 10.1007/s10549-016-3709-z 26907764

[54] Mungai F Verrone GB Pietragalla M Berti V Addeo G Desideri I. CT assessment of tumor heterogeneity and the potential for the prediction of human papillomavirus status in oropharyngeal squamous cell carcinoma. La Radiologia Med. (2019) 124:804–11. doi: 10.1007/s11547-019-01028-6 30911988

[55] Peng B Wang K Xu R Guo C Lu T Li Y. Preoperative computed tomography-based tumoral radiomic features prediction for overall survival in resectable non-small cell lung cancer. Front Oncol. (2023) 13:1131816. doi: 10.3389/fonc.2023.1131816 37207163 PMC10189057

