# Supplementary figures and images for: Machine learning-based integration of DCE-MRI radiomics for STAT3 expression prediction and survival stratification in breast cancer
Source: Front Immunol. 2025 Jun 25;16:1619186. doi: 10.3389/fimmu.2025.1619186 (PMC12237646; doi:10.3389/fimmu.2025.1619186)

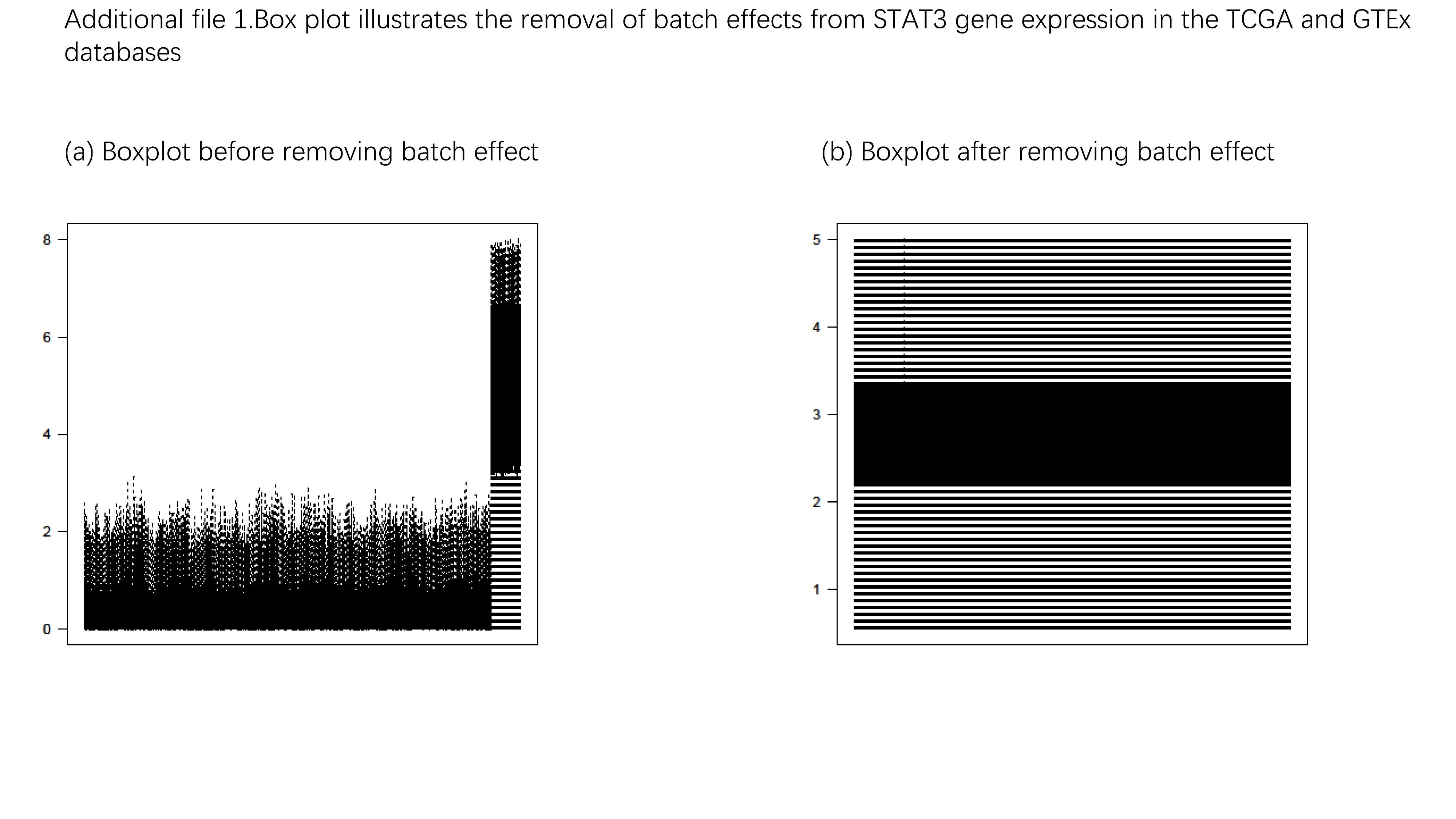

Supplement: Additional file 1 — Box Plot Illustrating the Removal of Batch Effects from STAT3 Gene Expression Data in the TCGA and GTEx Databases. (File format:.tif). [file Image1.tif]

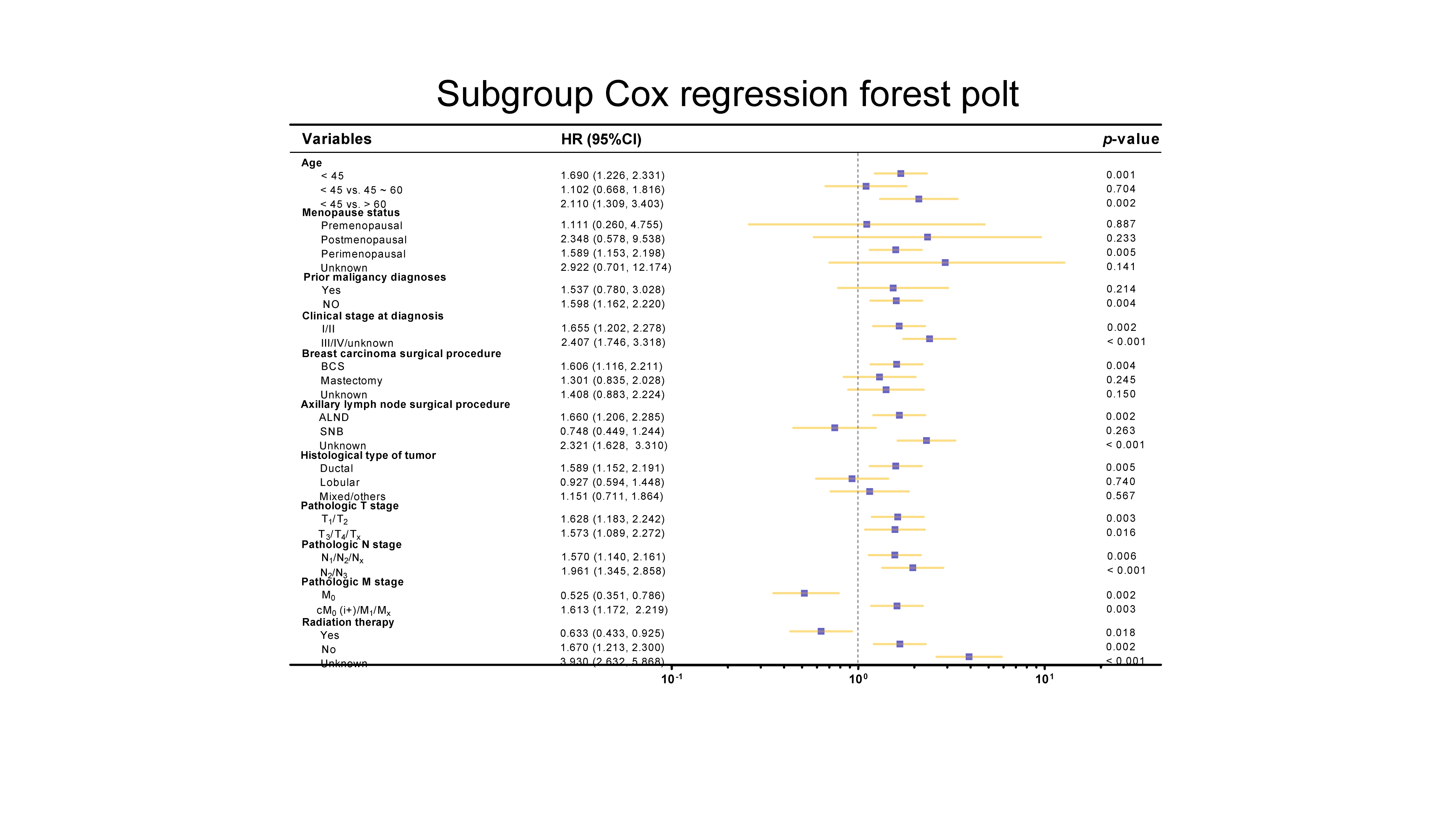

Supplement: Additional file 4 — Forest Plot of Subgroup Cox Regression Analysis. (File format:.tif). [file Image4.tif]

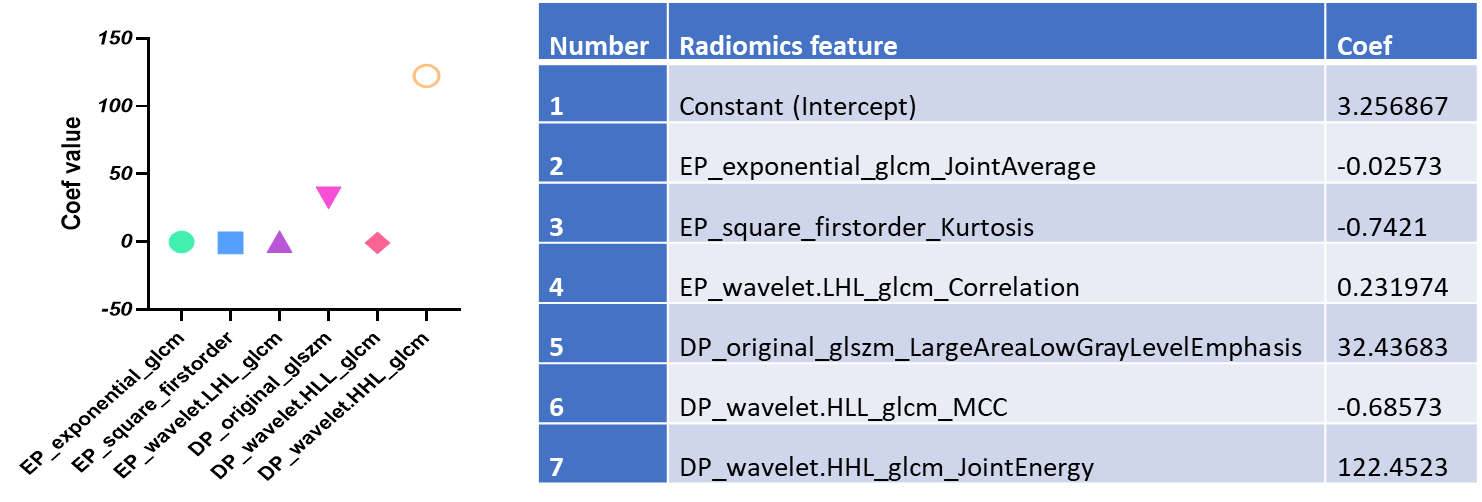

Supplement: Additional file 7 — Weighted Coef Values of Each Features. (File format:.docx). [file Table7.docx]

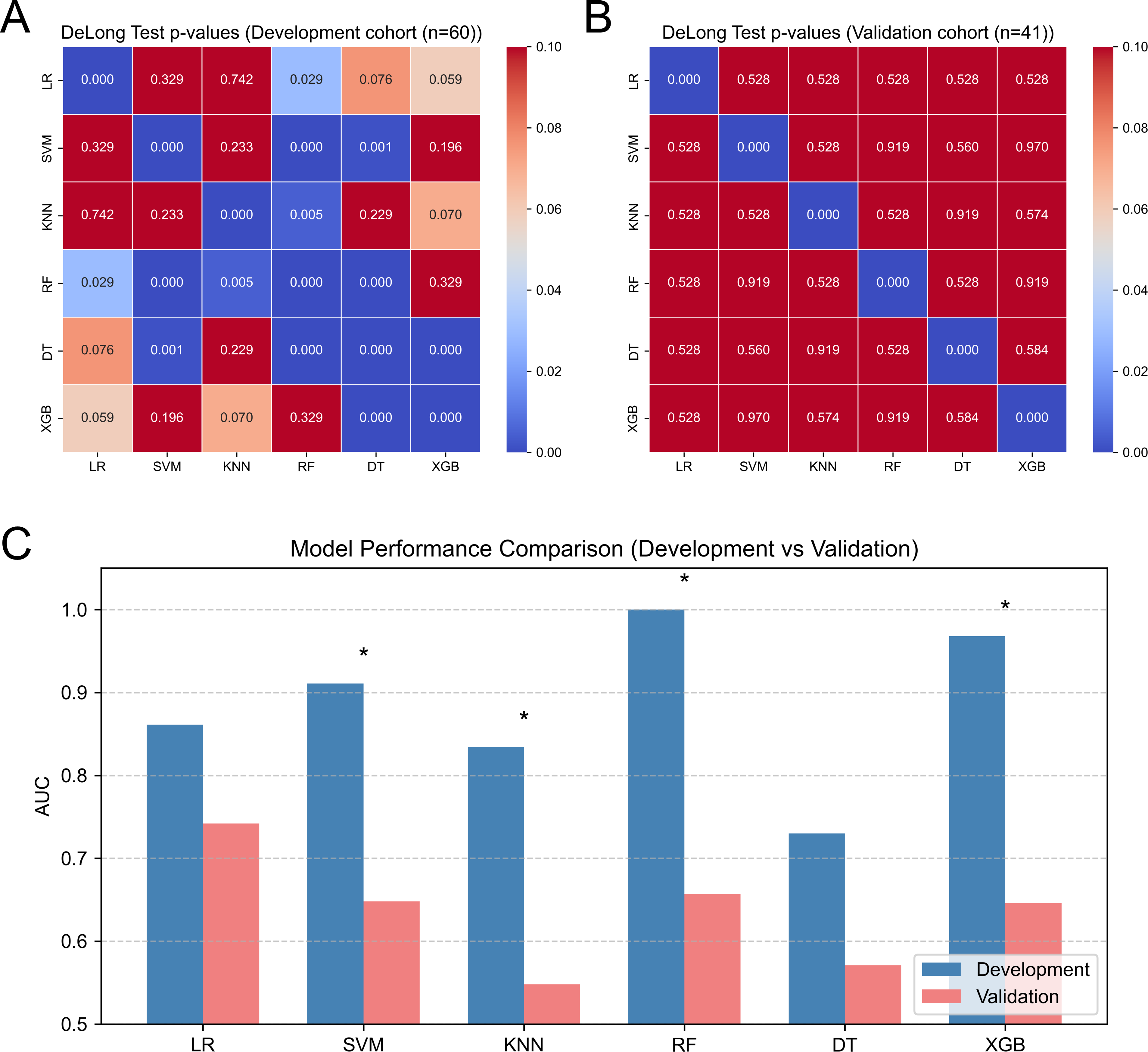

Supplement: Additional file 9 — DeLong’s test between models within and across development validation cohorts. (File format:.tif). [file Image9.tif]

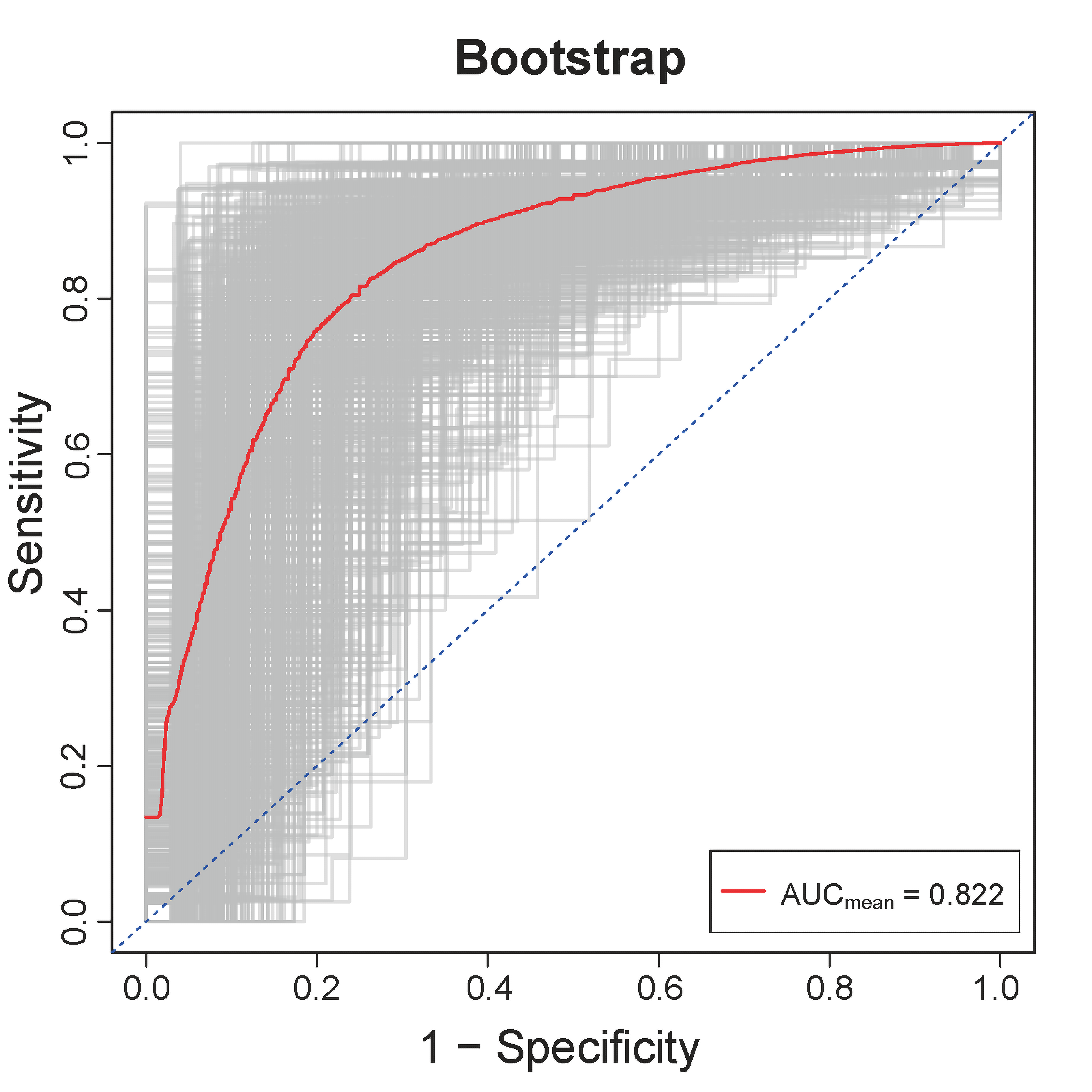

Supplement: Additional file 10 — ROC Curve Derived from Bootstrap Analysis. (File format:.tif). [file Image10.tif]
