# Supplementary material for: Machine learning-based integration of DCE-MRI radiomics for STAT3 expression prediction and survival stratification in breast cancer
Source: Front Immunol. 2025 Jun 25;16:1619186. doi: 10.3389/fimmu.2025.1619186 (PMC12237646; doi:10.3389/fimmu.2025.1619186)
Supplement: Additional file 3 — Hyperparameter optimization during machine learning models construction. (File format:.docx). [file Table3.docx]

**Additional file 3.** Hyperparameter optimization during machine learning models construction

| **Model** | **Networks Definition** | **Optimal Hyperparameters** |
| --- | --- | --- |
| LR | alpha = 0  lambda = 10^seq[-4, -1, length = 10] | alpha = 0  lambda = 0.01 |
| SVM | C = [0.1, 1, 2]  sigma = [0.05, 0.1] | C = 1  sigma = 0.1 |
| KNN | k = seq[5, 25, by = 5] | k = 10 |
| RF | mtry = max(2, floor(sqrt(ncol(train_set_lasso)))); splitrule = "gini"; min.node.size = c(3, 5) | mtry = sqrt(6), min.node.size = 5 |
| DT | cp = [0.01, 0.02, 0.05] | cp = 0.02 |
| XGBoost | n_estimators = [100, 200, 300]  max_depth = [3, 5, 7]  learning_rate = [0.01, 0.05, 0.1]  lambda = [1, 5, 10]  alpha = [0, 1, 5]  subsample = [0.6, 0.7, 0.8]  colsample_bytree = [0.6, 0.7, 0.8] | n_estimators = 200, max_depth = 5, learning_rate = 0.1, lambda = 5,  alpha = 1  subsample = 0.8, colsample_bytree = 0.7 |
