# Supplementary material for: Machine learning-based integration of DCE-MRI radiomics for STAT3 expression prediction and survival stratification in breast cancer
Source: Front Immunol. 2025 Jun 25;16:1619186. doi: 10.3389/fimmu.2025.1619186 (PMC12237646; doi:10.3389/fimmu.2025.1619186)
Supplement: Additional file 5 — Comparison of the Characteristics of the Development and Validation Cohorts. (File format:.docx). [file Table5.docx]

| Variables | Total  (n = 101) | Development cohort  (n = 60) | Validation cohort  (n = 41) | *p*-value |
| --- | --- | --- | --- | --- |
| **Age**  < 45  45 ~ 60  > 60 | 25 (24.8)  46 (45.5)  30 (29.7) | 16 (26.7)  26 (43.3)  18 (30.0) | 9 (22.0)  20 (48.8)  12 (29.3) | 0.826 |
| **Menopause status**  Premenopausal  Postmenopausal  Perimenopausal  Unknown | 39 (38.6)  54 (53.5)  6 (5.9)  2 (2.0) | 23 (38.3)  31 (51.7)  5 (8.3)  1 (1.7) | 16 (39.0)  23 (56.1)  1 (2.4)  1 (2.5) | 0.662 |
| **Prior malignancy diagnoses**  Yes  No | 98 (97.0)  3 (3.0) | 58 (96.7)  2 (3.3) | 40 (97.6)  1 (2.4) | 1.000 |
| **Clinical stage at diagnosis**  I/II  III/IV/Unknown | 87 (86.1)  14 (13.9) | 51 (85.0)  9 (15.0) | 36 (87.8)  5 (12.2) | 0.914 |
| **Breast carcinoma surgical procedure**  BCS  Mastectomy  Unknown | 30 (29.7)  36 (35.6)  35 (34.7) | 17 (28.3)  24 (40.0)  19 (31.7) | 13 (31.7)  12 (29.3)  16 (39.0) | 0.532 |
| **Axillary lymph node surgical procedure**  Axillary lymph node dissection  Sentinel node biopsy alone  Unknown | 40 (39.6)  36 (35.6)  25 (24.8) | 24 (40.0)  21 (35.0)  15 (25.0) | 16 (39.0)  15 (36.6)  10 (24.4) | 0.987 |
| **Histological type of tumor**  Ductal  Lobular  mixed/others | 85 (84.2)  12 (11.9)  4 (4.0) | 48 (80.0)  10 (16.7)  2 (3.3) | 37 (90.2)  2 (4.9)  2 (4.9) | 0.192 |
| **Pathologic T stage**  T1/T2  T3/T4/Tx | 97 (96.0)  4 (4.0) | 58 (96.7)  2 (3.3) | 39 (95.1)  2 (4.9) | 1.000 |
| **Pathologic N stage**  N0/N1/NX  N2/N3 | 88 (87.1)  13 (12.9) | 51 (85.0)  9 (15.0) | 37 (90.2)  4 (9.8) | 0.638 |
| **Pathologic M stage**  M0  cM0 (i+)/M1/Mx | 89 (88.1)  12 (11.9) | 53 (88.3)  7 (11.7) | 36 (87.8)  5 (12.2) | 1.000 |
| **Radiation therapy**  Yes  No | 71 (70.3)  30 (29.7) | 42 (70.0)  18 (30.0) | 29 (70.7)  12 (29.3) | 1.000 |
| **STAT3 expression**  High  Low | 64 (63.4)  37 (36.6) | 36 (60.0)  24 (40.0) | 28 (68.3)  13 (31.7) | 0.523 |
