# Supplementary material for: Machine learning-based integration of DCE-MRI radiomics for STAT3 expression prediction and survival stratification in breast cancer
Source: Front Immunol. 2025 Jun 25;16:1619186. doi: 10.3389/fimmu.2025.1619186 (PMC12237646; doi:10.3389/fimmu.2025.1619186)
Supplement: Additional file 8 — Description of selected radiomic features and their clinical relevance. (File format:.docx). [file Table8.docx]

**Additional file.** Description of selected radiomic features and their clinical relevance

| **Feature Name** | **Feature Class** | **Interpretation** | **Clinical / Biological Relevance** |
| --- | --- | --- | --- |
| EP_exponential_glcm_JointAverage | Texture (GLCM) | Average intensity of pixel pairs | Reflects tissue homogeneity; lower values may indicate tumor heterogeneity |
| EP_square_firstorder_Kurtosis | Intensity statistics | Peakiness of intensity distribution | Higher values suggest presence of outlier intensities (e.g., calcifications or necrosis) |
| EP_wavelet.LHL_glcm_Correlation | Wavelet-filtered texture | Linear dependency of neighboring pixels | Measures structural regularity; may correlate with tumor differentiation grade |
| DP_original_glszm_LargeAreaLowGrayLevelEmphasis | Size-zone (GLSZM) | Combination of large homogeneous areas with low intensity | Potential marker for necrotic regions or cystic components |
| DP_wavelet.HLL_glcm_MCC | Wavelet-filtered texture | Maximal correlation coefficient | Quantifies complex patterns in tumor architecture |
| DP_wavelet.HHL_glcm_JointEnergy | Wavelet-filtered texture | Uniformity of pixel pairs | Higher values indicate more uniform textures, potentially reflecting less aggressive phenotypes |
